# Comparative surgical resection of the ligamentum teres hepatis in a cadaveric model and a patient with ovarian cancer

**DOI:** 10.4274/jtgga.galenos.2018.2018.0135

**Published:** 2019-02-26

**Authors:** İlker Selçuk, Zehra Öztürk Başarır, Nurian Ohri, Bertan Akar, Eray Çalışkan, Tayfun Güngör

**Affiliations:** 1 Department of Gynecologic Oncology, Health Sciences University, Ankara Zekai Tahir Burak Woman’s Health Training and Research Hospital, Ankara, Turkey; 2 Department of General Surgery, Division of Surgical Oncology, Ankara University Faculty of Medicine, Ankara, Turkey; 3 Department of Obstetrics and Gynecology, Bahçeşehir University Faculty of Medicine, İstanbul, Turkey; 4 Department of Obstetrics and Gynecology, Okan University, WM Medical Park Hospital, Kocaeli, Turkey

**Keywords:** Pont hepatique, umbilical ligament, liver, ovarian cancer, cytoreduction

## Abstract

Resection of all tumor implants with the aim of maximal cytoreduction is the main predictor of overall survival in ovarian carcinoma. However, there are high risk sites of tumor recurrence, and the perihepatic region, especially the point where the ligamentum teres hepatis enters the liver parenchyma under the hepatic bridge (pont hepatique), is one of them. This video demonstrates the resection of the ligamentum teres hepatis both in a cadaveric model and in a patient with ovarian cancer.

## Introduction

The falciform ligament divides the liver into the right and left lobes on the antero-superior part of portoumbilical fissure where the ligamentum teres hepatis (umbilical ligament of liver/round ligament of liver) attaches to the visceral surface. Due to the distribution pattern of the portal vein and hepatic veins, the liver is divided into eight functional segments (1). The umbilical fissure exists between liver segments III and IVb, and the umbilical ligament lies there. The liver parenchyma over this structure varies in thickness, and in some patients the umbilical ligament will be completely visible, which allows broad exposure until its entrance into the liver. Paul Sugarbaker defined this parenchyma surrounding the umbilical ligament as the ‘pont hepatique/hepatic bridge,’ which creates a tunnel (2,3).

Mucinous ovarian or gastrointestinal carcinoma, appendiceal carcinoma, mesothelioma or a serous ovarian cancer may have a widely disseminated recurrence on the peritoneal surfaces. The complicated surgical anatomy of the liver and perihepatic tissues limits the easy detection of tumor implants; eventually, good exposure of the abdominal cavity is needed to excise all the visible tumor implants, especially on high-risk fields such as the end part of the ligamentum teres hepatis under the hepatic bridge (4). 

There is no risk of injuring any structures while cutting the hepatic bridge. However, if the ligament is deeply attached to the bottom of the liver parenchyma, while dissecting the end point, care should be taken not to damage the left hepatic artery or the left hepatic duct over the hepatoduodenal ligament, which is covered by the peritoneal lining of lesser sac (3,5). Routine resection of the ligamentum teres hepatis may increase morbidity (6); however, in patients with peritoneal carcinomatosis, the base of the ligamentum teres hepatis should be observed under the hepatic bridge because it is a continuation of peritoneal tissue.

This video consists a cadaveric surgical demonstration of ligamentum teres hepatis resection over the portoumbilical fissure and a live patient video of 56 years old woman who had a recurrent high-grade serous ovarian cancer with widespread peritoneal implants. There were tumor implants at the perihepatic region on the umbilical ligament, which were resected.

## Figures and Tables

**Figure 1:**
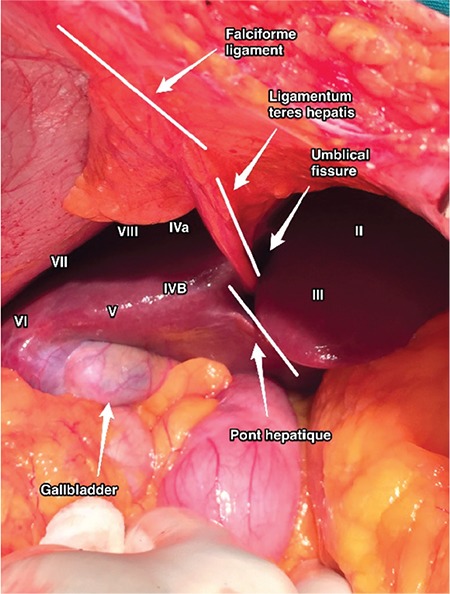
Localization of the pont hepatique and hepatic segmentation with the anatomic structures of the falciform ligament and ligamentum teres hepatis

**Figure 2:**
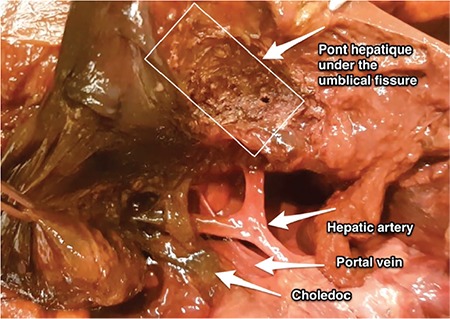
Cut end of the ligamentum teres hepatis over the liver parenchyma superior to hepatoduodenal ligament (choledoc, portal vein and hepatic artery)

**Figure 3:**
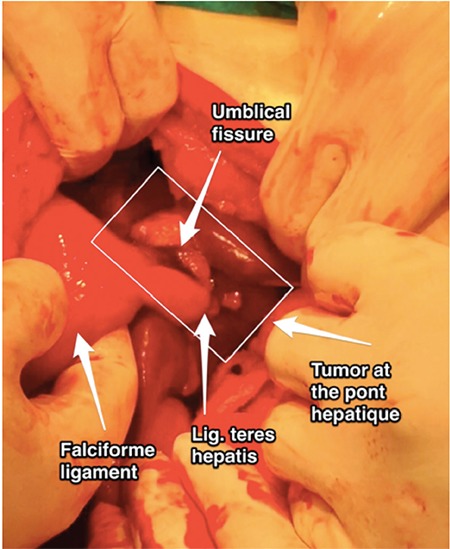
Tumor implants at the ligamentum teres hepatis and pont hepatique
